# Evaluation of ceftriaxone-sulbactam-disodium edetate adjuvant combination against multi-drug resistant Gram-negative organisms

**DOI:** 10.4102/ajlm.v9i1.991

**Published:** 2020-12-10

**Authors:** Shilpi Gupta, Mahadevan Kumar, Shelinder P.S. Shergill, Kundan Tandel

**Affiliations:** 1 Department of Microbiology, Military Hospital, Bhopal, India; 2 Department of Microbiology, Bharati Vidyapeeth University Medical College, Pune, India; 3 Department of Microbiology, Armed Forces Medical College, Pune, India; 4 Department of Microbiology, Command Hospital (Central Command), Lucknow, India

**Keywords:** ceftriaxone sulbactam, disodium edetate, multi-drug resistance, carbapenem-sparing

## Abstract

**Background:**

Multi-drug resistant (MDR) Gram-negative bacteria are an emerging threat, both in hospital and community settings. As very few antibiotics are effective against such infections, the need of the hour is a new antibiotic or drug combination which can overcome the effect of extended-spectrum β-lactamases (ESBL) and metallo β-lactamases (MBL). A new antibiotic combination of ceftriaxone, sulbactam and disodium edetate (CSE) has recently been proposed to tackle the MDR organisms.

**Objective:**

Our study was carried out to assess the susceptibility of ESBL- and MBL-producing Gram-negative organisms to CSE.

**Methods:**

The study was conducted in a tertiary-care hospital in Delhi, India, from February 2017 to June 2017. A total of 179 MDR (85 ESBL + 94 MBL) Gram-negative isolates from various clinical samples, identified by an automated system (Vitek 2) were tested against CSE using the Kirby-Bauer disc diffusion method. Susceptibility to CSE was recorded based on interpretative zone sizes of ceftriaxone as per 2017 Clinical and Laboratory Standards Institute guidelines.

**Results:**

The most common isolate was *Escherichia coli* (76/179; 42.4%) followed by *Klebsiella pneumoniae* (53/179; 29.6%) and *Acinetobacter baumanii* (27/179; 15.1%). The *in vitro* susceptibility of ESBL- and MBL-producing Gram-negative isolates to CSE was found to be 58/85 (68.2%) for ESBL and 37/94 (39.4%) for MBL.

**Conclusion:**

The *in vitro* susceptibility results obtained for CSE against ESBL-producing organisms is promising. It has the potential to emerge as a carbapenem-sparing antibiotic, active against ESBL-producing strains. Further clinical studies are required to establish the clinical efficacy of CSE against MDR pathogens.

## Introduction

Increased antimicrobial resistance of Gram-negative bacteria (GNB), both hospital- and community-acquired, is of great concern worldwide.^1^ According to the report of Global Antibiotic Resistance Partnership – India Working Group, the irrational and increased use of antibiotics, especially cephalosporins, in India has resulted in the emergence of multi-drug resistant (MDR) bacteria which were earlier known to be susceptible.^2^ The menace of the emerging threat has been realised by the Indian Government, hence it has called for effective action to address the increasing antimicrobial resistance. The Indian Ministry of Health and Family Welfare, in collaboration with the World Health Organization, formulated the National Health Policy 2017, which calls for an urgent need for standardisation of antibiotic usage guidelines to minimise the emergence of antimicrobial resistance. Thus, both organisations decided to tackle the issue as a priority collaborative work in 2018–2019.^3^

Despite increasing antibiotic resistance, the common therapy for treatment of GNB infections includes use of β-lactams with β-lactamase inhibitors, third and fourth generation cephalosporins and carbapenems.^4^ Production of β-lactamase enzymes is the most widespread mechanism of resistance adopted by GNB to counteract the effect of β-lactam antibiotics.^5,6^ Extended-spectrum β-lactamases (ESBLs) are usually plasmid-mediated β-lactamases and hydrolyse oxyimino group-containing β-lactam antibiotics.^7^ Metallo β-lactamases (MBLs) are a class of broad-substrate spectrum enzymes that also hydrolyse most β-lactam antibiotics, except monobactams.^8^ Other mechanisms that contribute to resistance are drug efflux systems, outer membrane protein changes, antibiotic-modifying enzymes and antibiotic-target modification.^9^ Carbapenems are used against the ESBL-producing organisms because of their stability against hydrolysis by ESBLs and broad-spectrum activity.^10^ However, with the emergence of carbapenem-hydrolysing enzymes, overexpression of efflux pumps and changes in outer membrane proteins, increases in carbapenem resistance have been reported among the members of enterobacteriaceae and non-fermenter GNB, such as the *Acinetobacter* and *Pseudomonas* group of pathogens.^11,12^

In India, the prevalence of ESBL and MBL producers among Gram-negative organisms range between 28% – 84% and 7.5% – 71%, respectively.^13,14^ The increasing resistance towards available antibiotics, as well as the lack of development of new antibiotics against GNB, could soon lead to the world experiencing the tough situations of the pre-antibiotic era with an increase in cases with untreatable infections. A newer approach to improving the efficiency of the existing antimicrobials is the use of antibiotic adjuvants. A novel antibiotic adjuvant entity of ceftriaxone, sulbactam with adjuvant disodium edetate (CSE) is being used in Indian hospitals against MDR infections.^15,16^ The antibiotic adjuvant entity is a combination of ceftriaxone plus sulbactam with disodium edetate and has undergone Phase III clinical trials under the aegis of the Central Drugs Standard Control Organisation, India.^17^ This study aimed to study the *in vitro* susceptibility to CSE of MDR Gram-negative organisms isolated in our centre. Thus, the present study aimed to study the *in vitro* susceptibility of ESBL- and MBL-producing GNB to CSE and to explore whether it could be utilised as a carbapenem-sparing drug.

## Methods

### Ethical considerations

Ethical clearance was obtained from the Institutional Ethics Committee, Army Hospital (Research and Referral), New Delhi, India (92/2016).

### Study design and setting

This cross-sectional study was conducted in the Department of Microbiology of a 900-bed, tertiary-care, super-speciality Army Hospital (Research and Referral), New Delhi, India from February 2017 to June 2017. Isolates were obtained from various clinical samples from both out- and inpatients received in the laboratory for bacterial culture from different clinical departments. Sample types included: urine, pus, cerebrospinal fluid, sputum, tissue from burn wound sites, endotracheal aspirate, semen, high vaginal swab and drains fluid.

### Microbiological processing

Samples were processed using conventional methods. Blood culture bottles were incubated in a fully automated blood culture system, the BacT/Alert 3D (bioMérieux SA, Marcy-l’Étoile, France). After obtaining a pure bacterial growth, isolate identification and antibiotic sensitivity testing were carried out on a Vitek 2 Compact (bioMérieux SA, Marcy-l’Étoile, France).

An MDR isolate was defined as a GNB strain that showed resistance to at least three different categories of antibiotics.^18^ A total of 85 ESBL- and 94 MBL-producing GNB were identified by phenotypic tests and confirmed by the Vitek 2 system for inclusion in the study. The confirmatory test for ESBL production was carried out using discs containing ceftazidime (30 *µ*g) alone, along with ceftazidime/clavulanic acid (30/10 *µ*g) discs. Similarly, cefotaxime (30 *µ*g) and cefotaxime/clavulanic acid discs (30/10 *µ*g) were also used. An increase in zone diameter of ≥ 5 mm around the clavulanate disk compared to the zone of inhibition for the ceftazidime and cefotaxime disk alone was used to confirm and isolate as positive for ESBL production as per Clinical and Laboratory Standards Institute guidelines.^19^ The modified Hodge test^19^ was used for isolates identified as carbapenemase-producing GNB strains. A 10 *µ*g meropenem disc was placed on a Mueller-Hinton agar plate previously inoculated with *Escherichia coli* American Type Culture Collection 25922 (the indicator organism). Afterwards, the test organisms were streaked out in a straight line, starting from the edge of the meropenem disc, for at least 20 mm – 25 mm length. The enhancement of growth of the indicator organism along the test organism’s streak line and zone of inhibition of the disk to form a cloverleaf appearance was considered as a positive indicator for carbapenemase production as per Clinical and Laboratory Standards Institute guidelines.^19^ For detection of class B carbapenemases (MBL), the double-disc synergy test using imipenem and imipenem plus ethylenediaminetetraacetic acid disc was done. An organism with a zone size difference of 7 mm between imipenem and imipenem plus ethylenediaminetetraacetic acid discs was considered to be an MBL-producing strain.^20^

**FIGURE 1:**
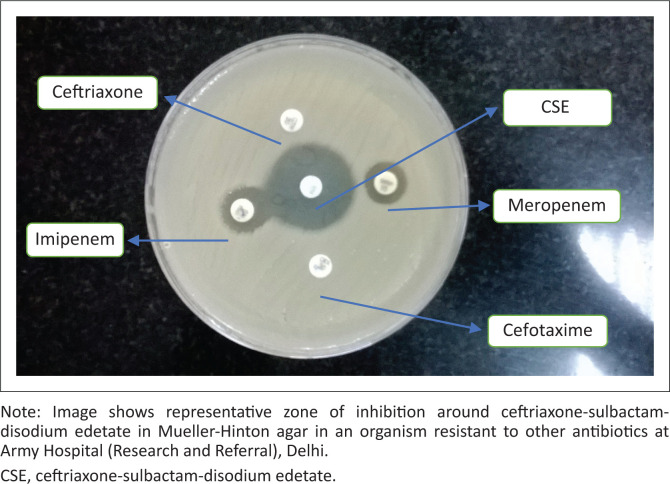
Zone of inhibition around the ceftriaxone-sulbactam-disodium edetate disc, Delhi, India, February 2017 to June 2017.

All 179 isolates were then further tested for antimicrobial susceptibility against CSE (Venus Medicine Research Centre, Baddi, Himachal Pradesh, India) by the Kirby-Bauer disc diffusion method on a Mueller-Hinton agar kept at 37 °C for 16 h – 18 h (Figure 1). Quality control of CSE antibiotic discs was done using *E. coli* American Type Culture Collection 25922 and a laboratory-characterised sensitive isolate of *Acinetobacter baumanii* (Strain no. AHRR1205/2017). Susceptibility of the tested organisms against this combination was reported as sensitive, intermediate or resistant based on the zone of inhibition mentioned for ceftriaxone as per Clinical and Laboratory Standards Institute guideline M100S27: Performance Standards for Antimicrobial Sensitivity Testing, 2017.^19^ The zone of inhibition around the disc was measured, and the organism was labelled as sensitive if the zone measured > 23 mm for enterobacteriaceae or > 21 mm for *Acinetobacter*, intermediate if 20 mm – 22 mm (enterobacteriaceae) or 14 mm – 20 mm (*Acinetobacter*), or resistant if < 19 mm for enterobacteriaceae or < 13 mm for *Acinetobacter*.^19^ Non-fermenters such as *Pseudomonas aeruginosa* and *Burkholderia cepacia* were not tested against CSE as these are known to be inherently resistant to ceftriaxone and there are no testing standards mentioned in Clinical and Laboratory Standards Institute guidelines.^19^

### Data analysis

Statistical analysis was done using Graph Pad, a free online software offering by founder Dr Harvey Motulsky (GraphPad Software, San Diego, California, United States). Associations between two factors were drawn through univariate logistic regression using the Fischer exact test. P-values of less than 0.05 were considered statistically significant.

## Results

**FIGURE 2:**
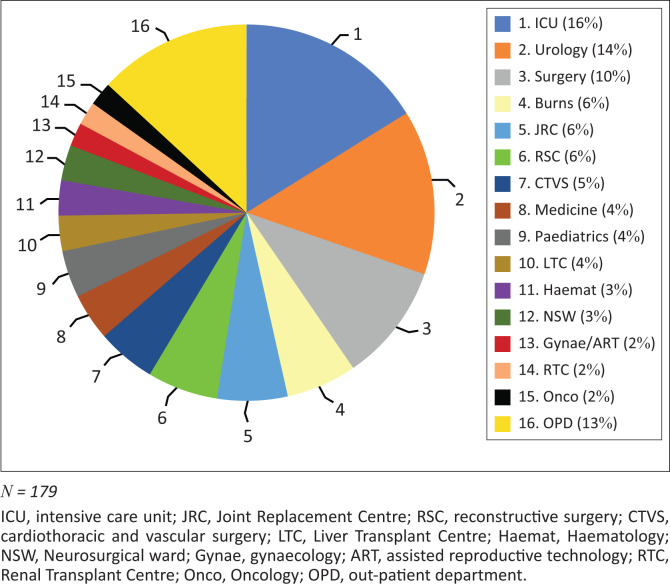
Ward distribution of samples included in the study at a hospital in Delhi, India, February 2017 to June 2017.

A total of 179 clinical isolates from 168 clinical cases (117 male patients and 51 female patients) were included in the study, with a mean patient age of 43.22 years (range: 4–85 years). Most isolates were from urine, followed by pus and blood specimens, and these accounted for 136 (75.9%) of the total isolates (Table 1). Of the included pathogens, 127 (70.9%) were isolated from inpatients, and 29 (16.2%) were isolated from patients in intensive care units (Figure 2).

**TABLE 1:** Prevalence of individual pathogens in various samples at a hospital in Delhi, India, February 2017 to June 2017.

Sample	Total no. of isolates (*n* = 179)	Isolates							
		*Escherichia coli* (*n* = 76)	*Klebsiella pneumoniae* (*n* = 53)	*Acinetobacter baumanii* (*n* = 27)	*Proteus mirabilis* (*n* = 13)	*Enterobacter cloacae* (*n* = 7)	*Citrobacter freundii* (*n* = 1)	*Serratia marcescens* (*n* = 1)	*Morgenella morganii* (*n* = 1)
Urine	84	49	22	2	8	1	1	1	0
Pus	34	10	5	12	4	3	0	0	0
Blood	18	6	10	2	0	0	0	0	0
Tracheal aspirate	11	1	2	7	0	1	0	0	0
Tissue	11	3	3	2	1	1	0	0	1
Sputum	6	1	3	2	0	0	0	0	0
CSF	5	1	4	0	0	0	0	0	0
Drain fluid	4	2	2	0	0	0	0	0	0
Semen	4	2	1	0	0	1	0	0	0
High vaginal swab	2	1	1	0	0	0	0	0	0

**Total**	**179**	**76**	**53**	**27**	**13**	**7**	**1**	**1**	**1**

CSF, cerebrospinal fluid

Among the isolated pathogens, *E. coli* (*n* = 76; 42.4%) was the most predominant followed by *Klebsiella pneumoniae* (*n* = 53; 29.6%) and *A. baumanii* (*n* = 27; 15%) (Table 1). Eighty-five (47.5%) of the tested isolates were ESBL producers and 94 (52.5%) were MBL producers. Fifty-eight (68.23%) of the ESBL producers (Table 2) and 37 (39.36%) MBL producers (Table 3) showed *in vitro* sensitivity towards CSE.

**TABLE 2:** Antibiogram for ceftriaxone-sulbactam-disodium edetate against extended-spectrum β-lactamases producing Gram-negative isolates at a hospital in Delhi, India, February 2017 to June 2017.

Organism	No. of isolates (*n* = 85)		CSE					
			Susceptible (*n* = 58)		Intermediate (*n* = 22)		Resistant (*n* = 5)	
	*n*	%	*n*	%	*n*	%	*n*	%
*Escherichia coli*	60	70.5	44	73.3	13	21.7	3	5.0
*Klebsiella pneumoniae*	13	15.3	7	53.8	6	46.2	0	-
*Acinetobacter baumanii complex*	2	2.4	1	50.0	1	50.0	0	-
*Proteus mirabilis*	3	3.5	2	75.0	0	-	1	25.0
*Enterobacter cloacae*	4	4.7	2	50.0	1	25.0	1	25.0
*Citrobacter freundii*	1	1.2	0	0.0	1	100.0	0	-
*Morganella morganii*	1	1.2	1	100.0	0	-	0	-
*Serratia spp.*	1	1.2	1	100.0	0	-	0	-

CSE, ceftriaxone, sulbactam and disodium edetate

**TABLE 3:** Antibiogram for ceftriaxone-sulbactam-disodium edetate against metallo β-lactamases producing Gram-negative isolates at a hospital in Delhi, India, February 2017 to June 2017.

Organism	No. of isolates (*n* = 94)		CSE					
			Susceptible (*n* = 37)		Intermediate (*n* = 36)		Resistant (*n* = 21)	
	*n*	%	*n*	%	*n*	%	*n*	%
*Escherichia coli*	16	17.0	5	31.3	7	43.8	4	6.7
*Klebsiella pneumoniae*	40	42.6	11	27.5	17	42.5	12	30.0
*Acinetobacter baumanii complex*	25	26.6	12	48.0	9	36.0	4	16.0
*Proteus mirabilis*	10	10.6	8	80.0	2	20.0	0	-
*Enterobacter cloacae*	3	3.2	1	33.3	1	33.3	1	33.3

CSE, ceftriaxone, sulbactam and disodium edetate

**FIGURE 3:**
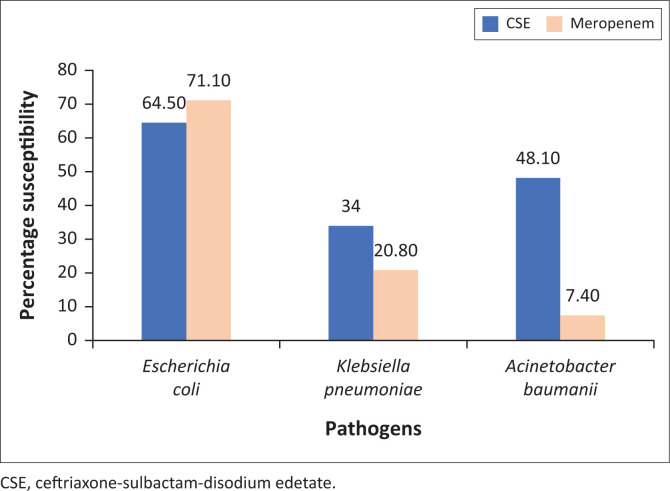
*In vitro* antibiotic susceptibility pattern for most common Gram-negative isolates against ceftriaxone-sulbactam-disodium edetate and Meropenem (*n* = 156) at a hospital in Delhi, India, February 2017 to June 2017.

Among the identified ESBL-producing GNB, 44 (73.3%) *E. coli* and 7 (53.8%) *K. pneumoniae* showed sensitivity towards CSE, while 51 (85%) *E. coli* and 11 (84.6%) *K. pneumoniae* showed sensitivity towards meropenem. The most common MBL-producing GNBs, *K. pneumoniae, A. baumanii* and *E. coli,* showed 27.5% (*n* = 11), 48% (*n* = 12) and 31.3% (*n* = 5) sensitivity, respectively, towards CSE and 2.5% (*n* = 1), 0% and 6.3% (*n* = 1) sensitivity, respectively, towards meropenem (data not shown). A statistically significant association was found when susceptibility to meropenem and CSE were compared (*p* < 0.001) in ESBL-producing *E. coli*. However, no statistically significant associations were seen when the CSE susceptibility pattern was compared to meropenem susceptibility patterns for other ESBL- and MBL-producing organisms. Twelve (48%) of the MBL-producing *A. baumanii* isolates which were resistant to meropenem showed susceptibility to CSE (data not shown). Multi-drug resistant *E. coli, K. pneumoniae* and *A. baumanii* showed 64.5% (*n* = 49), 33.9% (*n* = 18) and 48.1% (*n* = 13) susceptibility, respectively, towards CSE and 71.1% (*n* = 54), 20.8% (*n* = 11) and 7.4% (*n* = 2), respectively, against meropenem (Figure 3).

## Discussion

This study included only those MDR GNB isolates which were proven to be pathogenic, obtained from clinically established cases of infection. The majority of isolates were from urine samples (*n* = 84; 47%), followed by pus (*n* = 34; 19%) and blood (*n* = 18; 10%). The identified pathogens included *E. coli, K. pneumoniae* and *A. baumanii*, in decreasing order of prevalence. Similar distributions of isolates with similar specimen distribution have been reported by other studies.^21,22^ In the present study, *E. coli* (*n* = 49; 58.3%) was highly prevalent in urine samples, followed by *K. pneumoniae* (*n* = 22; 26.2%), indicating their significant role in urinary tract infections. Similar findings were also reported by Janifer et al. in Chennai, South India^23^ and Ruchika et al. in Gurgaon, Haryana, North India.^24^ *Klebsiella* spp. and *E. coli* are known to be the major causative agents for hospital-acquired infections. According to the National Health Service report of 2017 on Gram-negative bloodstream infections, *E. coli, P. aeruginosa* and *Klebsiella* spp. were responsible for 72% of all Gram-negative bloodstream infections, with *E.coli* accounting for 59% of the total cases.^25^ However, in the present study, *E. coli* and *K. pneumoniae* were implicated in 16 out of 18 (88.8%) Gram-negative bloodstream infection cases, with *K. pneumoniae* identified in the majority (10/18; 55.5%) of cases as compared to *E. coli* (6/18; 33.3%), which is in contrast to the National Health Service report.^25^ *Acinetobacter baumanii* is known to be an important pathogen in causing respiratory infections such as hospital-acquired pneumonia and bacteraemia, especially in intensive care patients, followed by skin and soft tissue infections and urinary tract infections.^26,27^ The present study has dissimilar findings in terms of isolation of *A. baumanii*, with maximum isolation from pus, because of skin and soft tissue infection cases (50%); followed by respiratory samples, because of respiratory infections (32.1%); and blood samples, because of bacteraemia (7.1%).

In the present study, 95 out of 179 (53.1%) MDR GNB isolates showed sensitivity to CSE *in vitro*. In similar studies conducted in the northern and western parts of India, higher susceptibility rates against CSE have been reported by Bhatia et al. (84% – 94%), Kumar et al. (81.9% – 94.74%), Bagga et al. (87.5% – 94.6%) and Sachdeva et al. (74.2% – 80.5%).^12,24,28,29^ The lower rate in our study compared to other studies could be because the isolates tested in the present study have been identified as ESBL or MBL producers, which was not specifically mentioned by other studies. The susceptibility of ESBL- and MBL-producing isolates to the CSE combination in the present study was from 68% for ESBL producers and 39% for MBL producers. The range is close to a similar study conducted in Mumbai, Maharashtra, India by Sahu et al.^30^

Because of increased clinical use of carbapenems against MDR GNB, our study also compared the efficacy of CSE against meropenem on Gram-negative isolates to use this new combination as a possible carbapenem-sparing drug. Carbapenems are considered to be the drug of choice for ESBL-producing GNB. The present study showed that 58 (68.2%) of the ESBL isolates were susceptible to CSE, of which *E. coli* susceptibility to CSE was statistically significant when compared with its susceptibility to meropenem (*p* < 0.001). The susceptibility profile to CSE of the three most predominant pathogens in our study, *E. coli* (49/76, 64.5%), *K. pneumoniae* (18/53, 34%) and *A. baumanii* (13/27, 48.1%) was comparable for *E.coli* (54/76, 71.1%) when compared to meropenem and high for *K. pneumoniae* (11/53, 20.8%) and *A. baumanii* (2/27, 7.4%). However, several authors from different parts of India (Haryana, western Uttar Pradesh, Gujarat) have reported significantly higher susceptibility to CSE when compared with meropenem.^24,28,31^ Our study showed comparable sensitivity among ESBL organisms to CSE (58/85, 68.2%) and meropenem (64/85, 75.3%), which implies that if CSE is tested against all ESBL isolates and they are found to be susceptible, CSE could be used as a drug of choice in place of carbapenems. Most of the MBL-producing organisms are resistant to carbapenems and the drug of choice for such isolates is polymixins. In the present study, 39.4% (34) of such isolates were susceptible to CSE; thus, CSE instead of polymixins could be considered as a therapeutic option in these cases.

One major concern is finding effective treatments for infection with *Acinetobacter* spp., which is now commonly isolated from critical areas in most of the hospitals worldwide.^24^ Our study found CSE to be effective in 48.1% of MBL-producing *A. baumanii* infections, which is a fair number, and use of CSE could be beneficial in such infections. A similar study conducted in Pune, India found that CSE was a superior antibiotic compared to other commonly used β-lactam antibiotics, including carbapenems, when tested against MDR GNB.^32^ A study conducted in Faridabad, Haryana, India, which evaluated the clinical use of CSE on patients, concluded that CSE should be used as a carbapenem-sparing drug and its combination with polymyxins can help to reduce mortality rates by successfully treating complicated MDR cases of intraabdominal, lower respiratory tract and urinary tract infections.^12^

### Limitations

The limitation of our study was the relatively small number of isolates tested. Larger sample size and diverse health facility level (primary to tertiary) studies would be required to rule out any referral bias that is expected in a tertiary-care hospital. Further, this study can be extended with application to clinical situations to have a clinico-microbiological correlation to guide clinicians for the judicious use of CSE against MDR pathogens.

### Conclusion

The results of this study show that CSE can potentially be effective among ESBL-producing bacteria, especially *E. coli*. The susceptibility of multi-drug resistant Gram-negative microorganisms to CSE suggests that CSE could be a good option as a carbapenem-sparing drug and also against some of the MBL-producing organisms.
